# The dignity of terminally ill children in pediatric palliative care: perspectives of parents and healthcare providers

**DOI:** 10.1186/s12904-023-01206-4

**Published:** 2023-07-05

**Authors:** Siyu Cai, Qiaohong Guo, Junyi Lin, Chanjuan Deng, Huijun Li, Xuan Zhou

**Affiliations:** 1 Center for Clinical Epidemiology and Evidence-based Medicine, Beijing Children’s Hospital, Capital Medical University, National Center for Children’s Health, Beijing, 100045 China; 2 School of Nursing, Capital Medical University, 10 Xitoutiao, Youanmen Wai, Fengtai District, Beijing, 100069 China; 3 Department of Nursing, Beijing Children’s Hospital, Capital Medical University, National Center for Children’s Health, Beijing, 100045 China; 4 Beijing Key Laboratory of Pediatric Hematology Oncology, Key Laboratory of Major Diseases in Children, Hematology Center, Beijing Children’s Hospital, National Key Discipline of Pediatrics (Capital Medical University), Ministry of Education, Capital Medical University, National Center for Children’s Health, 56 South Lishi Road, Xicheng District, Beijing, 100045 China

**Keywords:** Dignity, Pediatric Palliative care, Terminally ill children, Mainland China

## Abstract

**Background:**

The Chochinov Dignity Model was developed based on a cohort of adult patients with advanced cancer, but its role among dying children is not clear. This study aims to develop a model of dignity for children receiving pediatric palliative care based on the Chochinov Dignity Model.

**Methods:**

This is a descriptive qualitative study. Participants included a total of 11 parents and 14 healthcare providers who were recruited from a tertiary children’s hospital in Beijing and the Pediatric Palliative Care Subspecialty Group of the Pediatrics Society of the Chinese Medical Association using purposive sampling. Thematic framework analysis was used to analyze data.

**Results:**

The themes of the empirical Dignity Model were broadly supported in this study, but some themes were interpreted differently in the child population. Compared with the original model, some child-specific themes were identified including acknowledging regret, a sense of security, the company of important loved ones, realizing unfinished wishes, decent and dignified death, resolving family disputes, and fairness.

**Conclusions:**

This is the first study on Dignity Model for terminal children. Knowledge of children’s dignity can promote reflection of healthcare providers and caregivers regarding the values underlying their performance in pediatric palliative care, and develop certain practical interventions to strengthen children and their families’ sense of dignity at end of life.

**Supplementary Information:**

The online version contains supplementary material available at 10.1186/s12904-023-01206-4.

## Introduction

Children at the end of life experience significant physical, psychological, social, and spiritual suffering which leads to serious damage to dignity. Preserving patient dignity is one of the core values of palliative care [1, 2]. Chochinov et al. [3] developed an empirical Dignity Model based on a cohort of adult patients with advanced cancer which provided a perspective to understand the meaning of dignity for terminally ill patients and guidance for dignity-conserving care. Although the generalizability of the Dignity Model has been studied extensively in adults, few studies have explored and interpreted the model in the pediatric population, and its role among dying children is not clear.

The dignity of terminally ill children is significantly different from that of adults, and it is relevant to the physical, emotional, psychological, and socio-cultural developmental stages of children [4, 5]. Some researchers have explored the definition of a dignified death for children, but they have reported functional definitions rather than an empirically based dignity model [6–8]. Although Julião et al. [9] and Schuelke et al. [10] have adapted Dignity Therapy for children and adolescents to make it more developmentally appropriate, and other scholars have examined the feasibility of legacy-making interventions in children with cancer [11], none of these interventions were developed based on a dignity-relevant theoretical model for children with a terminal illness. As such, there does not yet exist a theoretical model that describes the construct of dignity for terminally ill children, based on which targeted, dignity-based psychological intervention can be developed for the pediatric population.

The research questions of this study are: what is the meaning of dignity for children with terminal illnesses, and what factors affect children’s and their parents’ perceptions of children’s dignity? This study aims to explore how parents and healthcare providers understand and define dignity for terminally ill children in order to develop a model of dignity for children receiving pediatric palliative care (PPC). This model will provide guidance for the dignity maintenance practices of healthcare providers, establish a framework for assessing the impact of care on children’s sense of dignity, and guide the development of dignity-based interventions.

## Method

### Design

This study used a qualitative descriptive design, a type of design that aims to comprehensively summarize targeted events, experiences, or perceptions through ‘‘low-inference’’ interpretation [12]. A basic qualitative descriptive design was considered the most appropriate method to meet the aim of this study: comprehensively describing the meaning of dignity for terminally ill children. The research method and its reporting followed the Consolidated Criteria for Reporting Qualitative Research (COREQ).

### Sample criteria

Participants included healthcare providers and parents of children receiving palliative care. Participants were recruited from a tertiary children’s hospital in Beijing and the PPC Subspecialty Group of the Pediatrics Society of the Chinese Medical Association using purposive sampling. Maximum variations were sought for the child’s diagnosis and age, as well as the healthcare provider’s professional group and working experience.

Parents were selected based on the following inclusion criteria: (1) their child was younger than 18 years of age; (2) the child was diagnosed with a terminal illness with an estimated prognosis of six months or less; and (3) the child was receiving palliative care. Inclusion criteria for healthcare providers were: (1) being a physician, nurse, or social worker; and (2) having more than three years of experience in caring for dying children. A total of 11 parents and 14 healthcare providers were invited and all agreed to participate.

Eligible participants were identified through clinical leads within the PPC team of the children’s hospital. Participants were invited by the two corresponding authors (XZ and QG) in person or via phone, and were given information about the purpose and process of the study.

### Data collection

Participants were individually interviewed either in person or online via Tencent meeting (an online meeting software) between March and August 2022 by SC (MD, female), QG (PhD, female), and XZ (MD, female), all of whom have received qualitative research training and have rich experience in qualitative interviews. Participants were given the interview guide prior to the interview and asked to complete a brief demographic questionnaire. Interviews were conducted either in a private and quiet room in the palliative care ward or in a private online meeting room; the interviewers remained neutral and nonjudgmental to the contents of what the participants discussed during the interviews. If participants had emotional reactions to difficult memories during the interview, the interview was paused until the participant was ready to continue, or healthcare providers on the ward were asked to provide emotional support to participants as needed. All interviews were audio-taped and transcribed verbatim, and personal information was collected before the interview and was irretrievably anonymized. Evidence of emotions was marked in the transcript.

The interview guide was developed based on the purpose of this study, and by referring to instruments previously used in other studies on similar topics. It included five questions: (1) What is your understanding of the dignity of dying children? (2) What did you do to protect the dignity of dying children? (3) What do you think of the significance of protecting the dignity of children in palliative care? (4) What factors do you think could affect the dignity of dying children? and (5) Is there any other information about the dignity of children you would want to share with us? A detailed interview guide can be found in the supplementary file 1.

### Data analysis

Thematic framework analysis [13] was performed using Microsoft Word and Excel. Analysis was both deductive, such that it was informed by the Chochinov Dignity Model, and inductive, with new emergent themes being explored that were not captured by the Dignity Model. Specifically, data were coded and analyzed as follows: (1) all audio recordings were transcribed verbatim; (2) a preliminary coding framework with themes and subthemes was developed by the research team based on the Chochinov Dignity Model; (3) two researchers (QG and SC) familiarized themselves with the data, recorded analytical notes and thoughts, and then independently coded the transcripts; (4) codes were summarized deductively into themes and subthemes in the coding framework; (5) codes and themes that arose from participants’ views but could not be captured by the coding framework were synthesized inductively as new emergent themes; (6) new themes were integrated into the coding framework, and themes in the coding framework with no supportive data were deleted, thus forming a new thematic framework explaining dignity of dying children; and (7) themes and subthemes of the dignity model for dying children were described and interpreted with supportive quotations. Saturation was reached at a conceptual level. To enhance the rigor and trustworthiness of the study, the coding framework was agreed upon by all authors. Codes, subthemes, and themes that emerged from data analysis were discussed regularly and constantly compared with potential deviant cases in regular team meetings; the research team discussed the final model and reached a consensus, and meanwhile returned the model to two research participants for validation. In addition, we used reflexive bracketing during the research process to reflect on our roles as researchers and consider the possible impacts of our background, experiences, and values on the research to avoid bias.

### Ethical considerations

The study was carried out in accordance with the Declaration of Helsinki. Ethical approval was provided by the Institutional Review Board at Capital Medical University (No. Z2023SY056). All participants signed the informed consent form, and were informed that they could refuse to participate or withdraw from the study at any time. Each participant was given a numerical identification number (e.g. [HCP 03]) to protect their personal information.

## Results

### Demographic characteristics

In total, 14 healthcare providers and 11 parents of 7 children were interviewed. The interviews lasted from 32 to 75 min. Demographic characteristics of healthcare providers and parents are shown in Tables 1 and 2, respectively. Healthcare providers included 1 male and 13 female participants, and their median age was 42 years old (range = 26–52). Parents included 5 males and 6 females, and their median age was 32 (range = 31–50).

**Table 1:** Characteristics of the healthcare providers (N = 14)

	Profession	Marital status	Whether have children	Academic degree	Professional rank	Years of working experience	Years of experience caring for dying children
HCP01	Nurse	Married	Yes	Bachelor	Nurse in charge	27	27
HCP02	Nurse	Married	No	Bachelor	Nurse in charge	8	3
HCP03	Nurse	Married	Yes	Bachelor	Associate professor nurse	30	20
HCP04	Nurse	Divorce	Yes	Bachelor	Professor nurse	32	30
HCP05	Nurse	Married	Yes	Bachelor	Nurse practitioner	32	32
HCP06	Nurse	Single	No	Bachelor	Nurse practitioner	5	3
HCP07	Nurse	Single	No	Bachelor	Nurse practitioner	5	5
HCP08	Nurse	Married	Yes	Bachelor	Nurse in charge	17	5
HCP09	Social worker	Married	Yes	Bachelor	Assistant social worker	7	7
HCP10	Social worker	Single	No	Master	Assistant social worker	3	3
HCP11	Physician	Married	Yes	Master	Chief physician	27	27
HCP12	Physician	Married	No	Master	Resident	3	3
HCP13	Physician	Married	Yes	Master	Attending physician	9	9
HCP14	Physician	Married	Yes	Master	Chief physician	24	24

**Table 2:** Characteristics of parents (N = 11) and their ill child (N = 7)

		Child’s gender	Child’s age	Child’s diagnosis
Family 01	Mother 01	Female	2	Medulloblastoma
Family 02	Mother 02	Female	9	Glioma
	Father 02			
Family 03	Mother 03	Male	2	Rhabdomyosarcoma
	Father 03			
Family 04	Mother 04	Male	2	Refractory intestinal obstruction
	Father 04			
Family 05	Father 05	Female	3	Leukemia
Family 06	Mother 06	Male	5	Neuroblastoma
Family 07	Mother 07	Female	5	Leukemia
	Father 07			

### Dignity model for dying children

**Fig. 1:**
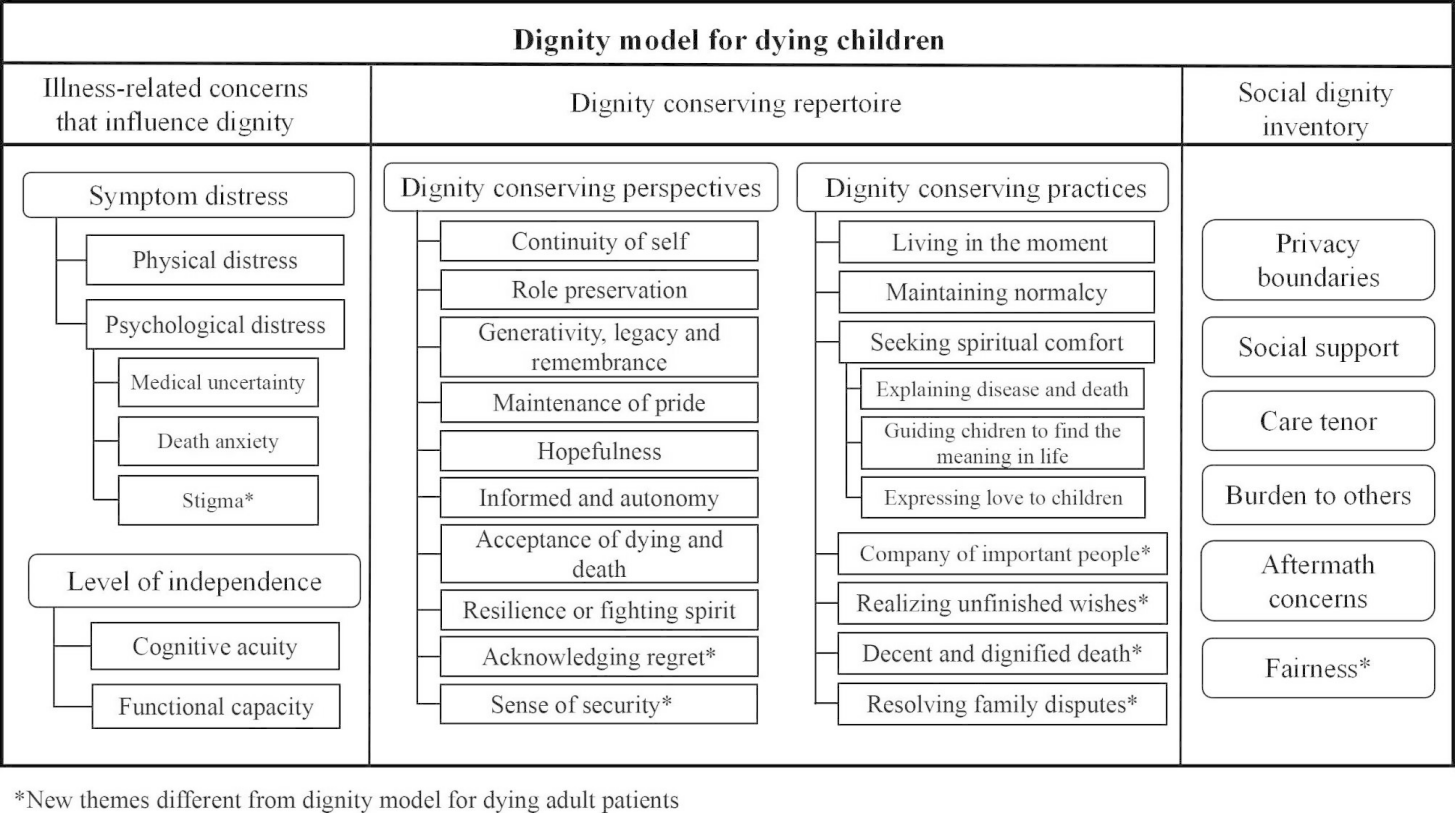
The dignity model for terminally ill children

Illness-related concerns, dignity-conserving repertoire, and social dignity inventory were the three major categories of children’s dignity (Fig. 1). Three factors affecting children’s and parents’ perceptions of children’s dignity were identified, including individual, familial, and cultural factors.

### Illness-related concerns that influence dignity

#### Symptom distress

**a. physical distress**

Both healthcare providers and parents stated that symptom control was fundamental to maintaining dignity. However, many children had difficulty expressing their symptom distress clearly. Therefore, detecting and assessing symptoms accurately was a major challenge to maintaining children’s dignity.

*“Although she [the child] won’t say it, she doesn’t want to be tortured by pain.” (Mother 01)*.

*“We need to make sure they are not tortured by pain or other painful symptoms. We must relieve the symptoms first. This is the most basic thing for them to regain their sense of dignity. No pain controls, no talk.” (HCP 11)*.

**b. psychological distress**

Psychological distress was described by participants in this study in three ways: medical uncertainty, death anxiety, and stigma. Children might experience fear, loneliness, anger, anxiety, and depression caused by medical uncertainty and death anxiety at the end of their lives. Participants noted that concealing prognosis from children would aggravate these negative emotions. It was difficult for children to obtain support because their psychological distress was often ignored by parents and healthcare providers. Stigma was the shame brought by the disease. The stigma of children and parents manifested as a refusal to let others know about the diseases and see the physical changes caused by the diseases.

*“People may discriminate against my kid and me. I don’t want that. This is about dignity.” (Mother 06)*.

*“When we can’t cure this child, I think it’s very important to respond to the child’s fears.” (HCP 10)*.

#### Level of independence

Although children had limited independence, healthcare providers in this study stated that a decline in independence still resulted in compromising the child’s dignity.

**a. cognitive acuity**

Participants stated that dignity is related to cognitive intactness. They found that some children expressed a willingness to tolerate a certain amount of pain in order to stay awake, while other children tended to choose comfort in the trade-off between cognitive sensitivity and comfort.

*“She [the child] lay unconscious in bed; this kind of life has no meaning.” (Mother 01)*.

*“Some children indicated that they don’t want to be too sleepy every day. They hope they can stay awake, even with a little bit of pain.” (HCP 12)*.

**b. functional capacity**

Disease led to impairment of children’s ability to carry out daily activities, which resulted in a fracture of their self-regard of competence and sense of dignity.

*“She had a urinary catheter when she was in the ICU, although she didn’t want to use it; she didn’t want to pee in the bed.” (Mother 07)*.

*“That child needs to spend a very long time to eat every day. But he would rather be hungry than have a nasal feeding tube.” (HCP 11)*.

### Dignity-conserving repertoire

#### Dignity-conserving perspectives

**a. continuity of self**

Poor body image caused by disease or treatment resulted in erosion of dignity of both patients and their families.

*“We hope there is no big change in his face. He may lose some weight, but don’t have obvious edema or large tumors on the face.” (HCP 13)*.

**b. role preservation**

Participants stated that the most important role in a child’s life was their role as a son or daughter. The pain of children losing their family role came from their inability to fulfill their filial duty to their parents. Another important role of children was that of the student. The sick children maintained their student role by keeping in touch with their classmates and teachers.

*“I heard a child said to his parents, ‘I’m sorry I can’t take care of you anymore, and I can’t be filial anymore’.” (HCP 11)*.

**c. generativity, legacy and remembrance**

Some children had existential distress and fear of being forgotten because they did not leave behind something transcendent of death. Different from adults, children did not accumulate accomplishments, contributions, wealth, and knowledge that could benefit others or spread to future generations. Leaving a sign that they had existed was important to the dignity of children. Healthcare providers mentioned that children created memories and legacies by taking photos and videos, making mementos, distributing belongings, writing letters, saying farewell, and so on.

*“His dad and I both want to donate his organs. At least it gives us something to miss.” (Mother 01)*.

*“Parents often take photos or videos for their children as a memento.” (HCP 13)*.

**d. maintenance of pride**

Because children lacked accomplishments, it was more difficult for them to maintain their sense of pride when they face serious diseases. Helping children find meaning and pride in their short lives could help conserve their dignity.

*“Her paintings were exhibited in our hospital. She may feel that she was needed. She thinks she’s great. She’s proud of herself.” (HCP 01)*.

**e. hopefulness**

Participants regarded that the hope at the end of life was not for the disease to be cured, but to maintain the enthusiasm and longing for life.

*“He still yearns for living in the world and appreciates the beauty of the world.” (Mother 06)*.

**f. informed and autonomy**

Some children wanted to be well-informed, allowing them to make decisions according to their autonomous wishes. Although children’s decision-making abilities are not fully developed, they should be regarded as a person with decision-making ability. Healthcare providers suggested that providing advance care planning services to children according to their decision-making ability and willingness helps to enhance children’s dignity. If children’s decisions are not valued and respected, they feel devalued and lack control.

*“She wanted to know the treatment. What kind of medicine to use. What will happen in the future … There was a five-year-old girl who made her own decisions about sedation.” (HCP 09)*.

*“I treat her like an adult. I told her why she needed treatment, and why we were here [in the hospice ward]. We asked if she wanted to continue treatment.” (Mother 07)*.

**g. acceptance of dying and death**

Healthcare providers mentioned that most children needed help to accept death, such as by meeting the communication needs of children for disease and death, and by providing life-and-death education for children. In addition, healthcare providers also found that young children generally had a good acceptance of death due to a lack of understanding of death.

*“A 12-year-old girl was very scared when she was dying. She said, ‘Mom, I don’t want to die, save me!’” (HCP 12)*.

**h. resilience or fighting spirit**

Participants regarded the end of life as a difficult journey. At this stage, children, parents, and PPC teams still need to maintain their fighting spirit. Stopping providing medical services for dying children was considered to be detrimental to the dignity of children.

*“We won’t let them feel that we don’t care about them anymore. We won’t ignore them just because they are about to leave.” (HCP 08)*.

**i. acknowledging regret**

Children had many unfinished businesses and their parents’ expectations for them could not be fulfilled, which can cause regrets. Healthcare providers and parents believed that respecting reality helped to maintain dignity; contrarily, refusing to acknowledge regret and creating a false sense of perfection would damage the dignity of children.

*“I think we should admit that he is in an unfinished state. He is only a teenager, and has never been in love, and has no sexual experience. I think it’s important for us to admit with him that some things couldn’t come true, rather than telling him that he already had a happy childhood.” (HCP 10)*.

*“If I made up a happy ending for her, she won’t believe it.” (Mother 07)*.

**j. sense of security**

Healthcare providers stated that children were more vulnerable to insecurity than adults at the end of life. The company of family members and a warming environment could provide a sense of security for children. They also stated that children were commonly afraid of hospitals and wanted to die at home, so it was suggested that the environment and atmosphere of the PPC ward should be like that of the home.

*“I think the most favorite and safest place for children is their home … They are very afraid of hospitals. Therefore, the environment of our hospice ward is quiet and warm, with fewer hospital elements in it.” (HCP 12)*.

### Dignity-conserving practices

**a. living in the moment**

Participants mentioned that living in the moment was a common coping strategy for children at the end of life. Children, especially younger children, were more concerned with the quality of life in the present rather than worrying about the future.

*“He cried bitterly during the treatment, but he stopped crying as soon as the treatment was over. He immediately became happy and started playing. He is very young, so he lives in the moment.” (Father 05)*.

**b. maintaining normalcy**

Healthcare providers reported that the dying children and their parents hoped to return to normal life. To achieve this goal, parents usually took their children home.

*“He wanted the last [of his] life to be the same as the state of his daily life … We should make sure he doesn’t lose his life.” (HCP 09)*.

**c. seeking spiritual comfort**

Due to age limitations, many children did not develop a comprehensive understanding of disease and death. Explaining diseases and death to children could help provide them with spiritual comfort. Healthcare providers found that the parents and children might have different understandings of death, which could lead to conflict and damage the dignity of children. Guiding children to find meaning in life was also helpful to maintain their dignity. Children had a relatively simple understanding of the meaning of life compared with adults. Expressing love to children was an important way to help children find meaning of life and enhance their sense of value.

*“His mother said that death means you would go to a particularly good place. After arriving at that place, you would be all right. You would no longer feel pain. You would become a bodhisattva and you could fly. Then the child was not afraid. The child said, “Oh, great, I’m going to be a bodhisattva!“ I think the spiritual support from the family is great. The child was satisfied and calm in the last days.” (HCP 12)*.

**d. company of important loved ones**

Loneliness could decrease the sense of dignity of children. Children hoped to be accompanied and comforted by important loved ones at the end of life.

*“Dying with dignity means having their parents and significant ones to be with him before he dies.” (HCP 07)*.

*“Our company is important for her dignity. No one’s company is better than that of parents.” (Mother 01)*.

**e. realizing unfinished wishes**

Helping children realize unfinished wishes and make up for regrets was an important way to enhance their dignity. Children’s wishes included doing what they like to do, eating their favorite food, receiving visits from significant ones, going home or any places they like, and so on.

*“We can help these children accomplish some unfinished wishes during their short life journey so that they can be [at] peace in [their] heart.” (HCP 08)*.

**f. decent and dignified death**

Washing the body, and wearing their favorite clothes for children after their death was an important way for family members and healthcare providers to maintain the dignity of children.

*“I think the greatest respect I give her is to try to erase all traces related to the hospital. We remove intravenous and other lines and tubes, clean her up, help her wear her favorite clothes, and dress her up.” (HCP 03)*.

*“I want to get the vein transfusion port out. I want my kids to pass away without these things on his body.” (Father 03)*.

**g. resolving family disputes**

Family disputes included parental disputes and parent-child disputes. These disputes affected the quality of life of children and damage their dignity. Resolving family disputes was an important way to maintain children’s dignity and inner peace.

*“He had a deep grudge against his father, and his heart was very painful. The nurses established a communication bridge to promote in-depth communication of the whole family and resolve family disputes. This communication was of great significance to the child’s dignity.” (HCP 14)*.

### Social dignity inventory

#### Privacy boundaries

Participants regarded privacy as an important aspect of children’s dignity, which consisted of physical privacy, space privacy, and communication privacy. Protecting the privacy of children was often ignored by parents and healthcare providers.

*“She [the child] had gender consciousness since she was three years old, and since then she knew she should cover her private parts. No matter what the situation was.” (Mother 07)*.

#### Social support

The social attributes of children are relatively simple compared with adults. Children’s social support usually came from family, friends, classmates, and teachers. In addition, the experience of long-term treatment enabled children to build close relationships with healthcare providers and other patients.

*“In addition to his family, some of his friends or teachers at school may be in close contact with him.” (HCP 08)*.

#### Care tenor

Although children are at a developmental stage, they should be recognized and treated as complete people. Healthcare providers stated that they protected the dignity of children by calling their names, introducing themselves, and explaining the care they would provide to children. Maintaining the dignity of children required respect for their inherent worth and uniqueness, including culture, values, spirituality, beliefs, experience, and perception of death. Healthcare providers should care for children with gentle attitudes and competent communication. In addition, because of the close relationship between children and their families, the dignity of family members should be considered as part of the dignity of children. Dignity maintenance in PPC was not limited to children but included the whole family.

*“Even if the child is very young, I respect him because he is an independent individual. For example, no matter how young the child is, I notify him when I want to give him a physical examination. If the child has a name, I call his name.” (HCP 11)*.

*“I mean, because you are you. First, you have your own experience. Second, you have your own way to experience. This is like an experiencing methodology … I think many adults are eager to export their own opinion to the child or force them to accept their opinions. I don’t think this is respectful.” (HCP 09)*.

#### Burden to others

Participants stated that although children needed to be raised and cared for, they were still afraid of becoming a burden to their families. To accommodate the child’s medical treatment, parents might quit their jobs, leave their hometown, be heavily mired in debt, and neglect to take good care of other children in the family.

*“He (the child) will think about whether his family is poor and whether he has spent a lot of money.” (HCP 01)*.

#### Aftermath concerns

Participants reported that children were less worried about the aftermath of their death, but they might want to participate in their own aftermath planning. Children were worried about the impact that death would have on those that were left behind, especially their parents. They might be deeply worried that their parents would grieve bitterly or even lose their courage to go on living. Participants found that many children wanted to help their parents to cope with loss after they are no longer living.

*“She planned her own aftermath. [She arranged] which toys she would take away and which would be left for her family.” (HCP 11)*.

#### Fairness

Participants found that children were extremely sensitive to fairness. They did not want to be treated either partially or ignored.

*“This child thinks that her mother has been taking care of her little brother. She is sick, but her mother didn’t take care of her. Other children are taken care of by their mothers. Why is she not?” (HCP 06)*.

### Factors affecting the children’s and parents’ perceptions of children’s dignity

#### Individual factors

Participants stated that children often demonstrate their need for dignity before they understand the connotation of dignity. Age, disease, and sense of self were important influencing factors of dignity perception. Participants noted that although children could perceive damage to their dignity, some could not accurately express their dignity needs due to their illness or developmental level. In addition, when the needs of children were inconsistent with their parents, the needs of children were often ignored.

*“A baby, or someone with severe brain damage, has no ability to express. It’s very difficult to know their dignity-related needs.” (HCP 02)*.

#### Familial factors

Family factors include parents’ education level and family culture. Family culture includes family environment and family inheritance. Families with high educational levels have higher needs for dignity maintenance. Dignity is a part of family culture and has been passed down from generation to generation. Children may not be fully aware of the indignities endured, but their families are. Therefore, some dignity needs might come from parents rather than the children themselves. In addition, conflict of dignity perceptions between parents and children could lead to dilemmas.

*“I think [maintaining dignity] is also related to the family environment of children. It comes down in one continuous line … If the whole family respects each other, everyone will care more about dignity.” (HCP 05)*.

#### Cultural factors

Cultural views on individual rights, autonomy, independence, and privacy impacted people’s perceptions of dignity at the end of life. Themes in the dignity model could be understood and applied differently in different cultures. For example, medical substitute decisions were seen in East Asian culture as protecting patients rather than depriving them of rights.

*“Respecting dignity is related to culture. Patriarchal culture is the mainstream culture in our country, which is bound to damage the subjectivity of the child.” (HCP 10)*.

## Discussion

To our knowledge, this is the first study on the Dignity Model for terminally ill children.

There has been a debate about whether children have a sense of dignity and the need to maintain dignity in children. Some researchers believe that dignity is an intrinsic and inalienable right of any human being [4]. People require a baseline level of respect as human beings, regardless of age or levels of consciousness [14]. Others consider that the acquisition of dignity is developmental, and children have a less-than-complete but developing sense of dignity [15]. This study found that parents and healthcare providers have a comprehensive understanding of the dignity of dying children and regard the maintenance of dignity as the central work of PPC. In addition, although age is an important factor affecting children’s dignity, many dignity themes considered to be exclusive to adults, such as a desire for independence and privacy, are in fact universal themes found across different age groups of children.

Dignity is a construct rooted in society, culture, beliefs, and life experience [16]. This study found that themes of illness-related concerns are basically the same between children and adults, but there are differences in dignity conserving repertoire and social dignity inventory.

Dignity Therapy is an intervention developed based on the Dignity Model intending to support the following themes: generativity, continuity of self, role preservation, maintenance of pride, hopefulness, aftermath concerns, and care tenor [11]. This study finds that these themes were interpreted in different ways in children’s dignity compared to adult dignity, indicating that interventions such as Dignity Therapy developed from the dignity model should be adapted for children. Dying children are eager to explore the meaning of life and self-value, and want to know they will not be forgotten [17]. This could be due in part to children’s lack of life experiences and developmental understanding of death [10, 18, 19]. Dignity Therapy could help children to create memories, confirm they are loved and will be remembered, and look for the meaning and value of life [11]. Previous studies have shown that the effects of legacy-making seemed to span across all pediatric age groups [20]. Therefor, dignity therapy could be a promising intervention for children in PPC after adaptation.

Sense of control is central to the maintenance of dignity. Children have different experiences from adults in controlling their lives and their bodies [15]. Mayall [21] proposed power as a force that structures a child’s relationship with adults who can affect their autonomy and subdue their voices in society; as such, realizing children’s autonomous function based on their cognitive abilities and willingness is an important way to enhance children’s dignity. Supporting children to participate in advance care planning facilitates their ability to maintain control over their personal affairs and care determinations [22, 23]. Not all children have the same degree of cognitive capacity and the same interest in making decisions about their care [24]. It is necessary to assess the extent to which a child has the capacity and desire to exercise autonomy [24, 25].

Being informed and exercising autonomy were described as some of the dignity-conserving perspectives for dying children in this study and are considered basic rights for human beings in western countries. However, the principle of beneficence has priority over the principle of autonomy in Chinese Confucian culture [26] and is influenced by the family-oriented and patriarchal culture [27]. Most parents make surrogate decisions for dying children, which was seen as a protective behavior, but to some extent could compromise the dignity of children [28]. Therefore, correctly dealing with cultural conflicts to achieve a balance between autonomy and beneficence should be considered while providing dignity-conserving care for children with Chinese cultural backgrounds.

### Implications for practice

Developing a dignity model for children receiving PPC is of great significance for practice, research, and education. The model can provide healthcare providers with guidance to maintain the dignity of children, and allow healthcare providers to reflect on the values underlying their performance in PPC. It can inform research looking to develop practical interventions to enhance the sense of dignity for children receiving PPC. The model can also be incorporated into medical education to raise the awareness of medical students in preserving patient dignity in their future clinical practice.

### Limitations and future research directions

This study reflects the perspective of Chinese healthcare providers and parents of terminally ill children. While their perceptions provide insights into children’s dignity at the end of life, views of dignity will vary based on factors such as region, culture, and religion. Therefore, it is important to understand the views of people in different regions concerning the dignity of children at end of life. In addition, this study focused on the dignity of children with a cancer diagnosis; further studies may focus on children who have other terminal diagnoses.

## Conclusion

The themes of the empirical Dignity Model are broadly supported in this study, but some themes are interpreted differently in the child population. Compared with the original model, some child-specific themes were identified, including acknowledging regret, a sense of security, the company of important loved ones, realizing unfinished wishes, decent and dignified death, resolving family disputes, and fairness.

## Electronic supplementary material

Below is the link to the electronic supplementary material.

Supplementary Material 1

## Data Availability

The datasets generated and/or analysed during the current study are not publicly available due we do not have permission from either the Research Ethics Board at the university or consented research participants to release or share the data but are available from the corresponding author on reasonable request.

## Abbreviations

PPC: pediatric palliative care
COREQ: Consolidated Criteria for Reporting Qualitative Research
