# Supplementary material for: The dignity of terminally ill children in pediatric palliative care: perspectives of parents and healthcare providers
Source: BMC Palliat Care. 2023 Jul 5;22:86. doi: 10.1186/s12904-023-01206-4 (PMC10320906; doi:10.1186/s12904-023-01206-4)
Supplement: Supplementary file 1 — Supplementary Material 1 [file 12904_2023_1206_MOESM1_ESM.docx]

INTERVIEW GUIDE

1. **Interview guide for parents**

(1) What is your understanding of the dignity of the child?

- What is your understanding of the dignity of the child when he/she is terminally ill?

(2) What do you think is the most important thing for the child at the moment?

(3) What do you think of the significance of protecting the dignity of children in palliative care?

(4) What factors do you think could affect the child's dignity?

- How does family affect the child's dignity? Any examples?

- How do illness and treatment affect the child's dignity? Any examples?

- How do healthcare providers affect the child's dignity? Any examples?

(5) What did you and your family do to protect the child's dignity? Any examples?

(6) What did the healthcare providers do to protect the child's dignity during treatment and care? Any examples?

(7) Is there any other information about the dignity of children you would want to share with us?

1. **Interview guide for healthcare providers**

(1) What is your understanding of the dignity of dying children? What do you think is a "dignified death" in pediatric palliative care?

(2) How do you evaluate the dignity needs of children and their parents?

(3) What do you think of the significance of protecting the dignity of children in palliative care?

(4) What factors do you think could affect the dignity of dying children?

- How does the family affect the children's dignity? Any examples?

- How do illness and treatment affect the children's dignity? Any examples?

- How do healthcare providers affect the child's dignity? Any examples?

1. What did the healthcare providers do to protect the child's dignity during treatment and care? Any examples?
2. Is there any other information about the dignity of children you would want to share with us?
